# Self-Management in Anxiety and Depression: A Psychometric Evaluation of a Questionnaire

**DOI:** 10.3389/fpsyg.2021.694583

**Published:** 2021-11-15

**Authors:** Esther Krijnen-de Bruin, Stasja Draisma, Anna D. T. Muntingh, Aagje Evers, Annemieke van Straten, Henny Sinnema, Jan Spijker, Neeltje M. Batelaan, Berno van Meijel

**Affiliations:** ^1^Psychiatry, Amsterdam Public Health Research Institute, Amsterdam UMC, Vrije Universiteit Amsterdam, Amsterdam, Netherlands; ^2^GGZ inGeest Specialized Mental Health Care, Amsterdam, Netherlands; ^3^Department of Health, Sports & Welfare, Research Group Mental Health Nursing, Inholland University of Applied Sciences, Amsterdam, Netherlands; ^4^Department of Clinical Neuro and Developmental Psychology, Faculty of Behavioral and Movement Sciences, Amsterdam Public Health Research Institute, Vrije Universiteit Amsterdam, Amsterdam, Netherlands; ^5^Institute for Nursing Studies, Utrecht and General Practice Linschoten, HU University of Applied Sciences, Utrecht, Netherlands; ^6^Expertise Center Depression Pro Persona Mental Health, Radboud University Nijmegen, Nijmegen, Netherlands; ^7^Parnassia Psychiatric Institute, Parnassia Academy, The Hague, Netherlands

**Keywords:** self-management, anxiety, depression, questionnaire, psychometric properties, exploratory factor analysis, confirmatory factor analysis

## Abstract

**Objective:** To examine the underlying factor structure and psychometric properties of the Assessment of Self-management in Anxiety and Depression (ASAD) questionnaire, which was specifically designed for patients with (chronic) anxiety and depressive disorders. Moreover, this study assesses whether the number of items in the ASAD can be reduced without significantly reducing its precision.

**Methods:** The ASAD questionnaire was completed by 171 participants across two samples: one sample comprised patients with residual anxiety or depressive symptoms, while the other consisted of patients who have been formally diagnosed with a chronic anxiety or depressive disorder. All participants had previously undergone treatment. Both exploratory (EFA) and confirmatory factor analyses (CFA) were conducted. Internal consistency and test–retest reliability were also assessed.

**Results:** Both EFA and CFA indicated three solid factors: Seeking support, Daily life strategies and Taking ownership [Comparative Fit Index = 0.80, Tucker Lewis Index = 0.78, Root Mean Square Error of Approximation = 0.09 (CI 0.08–1.00), Standardized Root Mean Square Residual = 0.09 (χ^2^ = 439.35, df = 168)]. The ASAD was thus reduced from 45 items to 21 items, which resulted in the ASAD-Short Form (SF). All sub-scales had a high level of internal consistency (> α = 0.75) and test–retest reliability (ICC > 0.75).

**Discussion:** The first statistical evaluation of the ASAD indicated a high level of internal consistency and test–retest reliability, and identified three distinctive factors. This could aid patients and professionals’ assessment of types of self-management used by the patient. Given that this study indicated that the 21-item ASAD-SF is appropriate, this version should be further explored and validated among a sample of patients with (chronic or partially remitted) anxiety and depressive disorders. Alongside this, to increase generalizability, more studies are required to examine the English version of the ASAD within other settings and countries.

## Introduction

Anxiety and depressive disorders are among the most prevalent mental health disorders across the globe (World Health Organization, 2016). Many patients experience a chronic course of symptoms, with chronicity ranging from 24.5% for depressive disorders to 41.9% for anxiety disorders (Penninx et al., 2011). Anxiety and depressive disorders have been found to often coincide. Out of those patients that reach remission, approximately 57% experience a relapse within the first four years (Scholten et al., 2016). Both the chronicity of these disorders and the frequency of relapses increase the overall burden of disease (Keller, 2006; Penninx et al., 2011), and are associated with a decreased quality of life (Alonso et al., 2004). Alongside the significant burden that it places on patients and their relatives, anxiety and depression also cause a tremendous economic burden worldwide (Walker et al., 2015).

The chronic and recurrent nature of anxiety and depression requires the adoption of a chronic disease approach (Collins et al., 2011). The use of self-management skills are generally considered to be essential in managing a chronic disease (Grypdonck, 1999; Lorig and Holman, 2003), which is why it constitutes a key part of the chronic care model (Bodenheimer, 2002). Self-management has been defined in manifold ways. Barlow et al. (2002) define it as “the individual’s ability to manage the symptoms, treatment, physical and psychosocial consequences and life style changes inherent in living with a chronic condition.” According to Lorig and Holman (2003), the six core self-management skills, from a patient’s perspective, are: problem solving, decision-making, resource utilization, the formation of a patient-provider partnership, action planning and self-tailoring. With respect to chronic conditions, self-management pertains to practices that patients themselves employ in order to manage their symptoms, avoid relapse, optimize their well-being (Barlow et al., 2002; Lorig and Holman, 2003), and, ultimately, improve the quality of their lives. Notwithstanding the role of the patient, professionals also have an important role to play in self-management promotion (Lorig and Holman, 2003).

In recent years, there has been an emergent focus on recovery in mental health care (Happell, 2008; Casey and Webb, 2019). Recovery is not only focused on reducing symptoms, but also signals “a movement toward health and meaning rather than avoidance of symptoms” (Clarke et al., 2012). From this perspective, recovery also encompasses personal and functional recovery. Manifold studies have acknowledged that self-management can be critically important in recovering from mental health disorders, due to their often chronic nature (Young and Ensing, 1999; Deegan, 2005; Sterling et al., 2010). There is increasing support for the assumption that the promotion by professionals in (mental) health care of patients’ self-management skills deserves more attention through the systematic development of self-management interventions that are highly tailored to self-management abilities of individual patients. For this purpose, more clarity is required about the concept of self-management and its operationalization, this in general and with respect of specific patient groups (Barlow et al., 2002; Lorig and Holman, 2003).

Extant literature has highlighted that there is a complex interaction between anxiety and depressive disorders and the use of self-management skills. For example, prior research has shown that the ability of patients with anxiety and depressive symptoms to perform self-management skills is influenced by the level of their symptoms, and that more severe anxiety and depressive symptoms lead to a decrease in self-management activities (Orem, 2001). Research has also demonstrated that the effective use of self-management strategies by patients suffering from anxiety or depression can reduce the severity of the symptoms for these disorders (Levitt et al., 2009). Hence, although depression and anxiety likely reduce patients’ ability to utilize self-management strategies, if self-management strategies are utilized they are likely to decrease patients’ anxiety and depressive symptoms.

There are a number of studies that have investigated the range of self-management strategies utilized by patients with anxiety and depressive disorders, which are often categorized into clusters (van Grieken et al., 2014, 2015; Chambers et al., 2015; Villaggi et al., 2015). The clusters that are most often described are: Engaging in activities (Chambers et al., 2015; van Grieken et al., 2015; Villaggi et al., 2015), Creating a routine (Chambers et al., 2015; Villaggi et al., 2015), and Seeking professional treatment (van Grieken et al., 2014; Villaggi et al., 2015). Engaging in activities mainly focuses on staying active, while creating a routine pertains to having a sense of structure and routinely planning activities. To the best of our knowledge, there is only one study available that psychometrically evaluates a self-management questionnaire for patients with anxiety, depressive and bipolar disorders, namely the Canadian developed Mental Health Self-Management Questionnaire (MHSQ; Coulombe et al., 2015). Given the potential cross-cultural differences in self-management (Shimizu and Paterson, 2007), not to mention that the MHSQ can be considered incredibly extensive (64 items), there was a need to develop, and subsequently psychometrically assess, a Dutch questionnaire that measured the self-management strategies employed by patients with anxiety and depressive disorders.

Consequently, Zoun et al. (2016) constructed a valid questionnaire by using a sample of Dutch patients with chronic anxiety and depressive disorders, which is called the Assessment of Self-management in Anxiety and Depression (ASAD). This questionnaire contains 45 self-management strategies relating to coping with a chronic anxiety and/or a depressive disorder. However, this questionnaire has hitherto not been psychometrically examined. Psychometric examinations can be carried out by conducting exploratory factor analysis (EFA) and confirmatory factor analysis (CFA). These statistical techniques reveal and confirm the underlying factor structure of a questionnaire, which results in either one or multiple factors explaining the common variance. Moreover, EFA and CFA are also statistical reduction techniques, which can help to determine if the items included in the questionnaire can be reduced, without significantly reducing the level of precision. Simply put, reducing the number of items greatly simplifies the survey administration, which, in turn, makes it less burdensome for patients.

The present study aims to gain insight into the underlying factor structure and psychometric properties of the ASAD, by using two samples of Dutch patients with either partially remitted or chronic anxiety and/or depressive disorders. Furthermore, this study examines whether the number of items in the ASAD can be reduced.

## Materials and Methods

### Study Design and Participants

This is a cross-sectional study that uses data from two separate studies: the GET READY study (Krijnen-De Bruin et al., 2019) and the SemCAD study (Zoun et al., 2016). These two studies were combined to increase the sample size and to assess the ASAD among patients who display the complete range of symptom severity, from mild to severe symptoms. As such, our sample is a good representation of patients who have been treated for anxiety or depressive disorders. An exploratory factor analysis (EFA), followed by a confirmatory factor analysis (CFA) were conducted to explore the underlying factor structure of the ASAD. Also, internal consistency, test–retest reliability and discriminant validity were tested.

#### Sample 1: GET READY Study

Data from the GET READY study was collected as part of an observational cohort study focused on relapse prevention. The participants in the GET READY study were adults who had undergone treatment for an anxiety and/or a depressive disorder within mental healthcare services in the Netherlands in the prior 2-year period, and who were subsequently in full or partial remission. Remission was defined by a score of ≤29 on the Beck Anxiety Inventory (BAI), and a score of ≤38 on the Inventory of Depressive Symptomatology-Self Report (IDS-SR). While measurements of the ASAD took place at baseline and after a 9-month period, only the baseline data was used for this study. A sub-sample completed the ASAD again 2 weeks after the baseline questionnaire, for test–retest measurements.

Fifty-four mental health professionals working in primary care practices throughout the Netherlands were recruited *via* telephone, letters, advertisements on websites, and through the researchers’ professional networks.

Patients were recruited by the mental health professionals at the end of their treatment, who provided brief information about the study. If patients were interested in participating, then the mental health professional asked consent from the patient to provide their contact details to the researchers. Next, consenting patients were contacted by the researchers and received additional information. This data was collected between April 2017 and November 2018. Further details on the GET READY study have been published previously (Krijnen-De Bruin et al., 2019).

#### Sample 2: SemCAD Study

The data from the SemCAD study was collected as part of a randomized controlled trial. The participants in the SemCAD study were adults who had been diagnosed with a chronic anxiety and/or depressive disorder, and had undergone treatment in outpatient specialist mental healthcare services in the Netherlands for at least a 2-year period, but who had yet to achieve recovery. The clinicians considered them to be treatment-resistant patients.

Participants were recruited from twelve Dutch specialized outpatient mental health care services for adults with anxiety and depressive disorders. Patients eligible for participating in the SemCAD study (Zoun et al., 2016) were asked to participate by their community psychiatric nurse. This nurse informed the patient about the study and provided an information letter to take home. After inclusion, patients received the baseline questionnaire. After administering this questionnaire, the patient was allocated to the experimental or control group of the trial.

The measurements took place at baseline (T0), 6 months (T1), 12 months (T2), and 18 months (T3). Given that the ASAD and Patient Health Questionnaire-9 (PHQ-9)/BAI were not collected at the same time, PHQ-9 and BAI data was used from T2, as this was the closest to the assessment of the ASAD. A detailed description of the SemCAD study has also previously been published (Zoun et al., 2016).

Written informed consent was obtained from all participants, and a Medical Ethics Committee approved both study protocols (Zoun et al., 2016; Krijnen-De Bruin et al., 2019). Given that the ASAD was developed for and with patients with chronic anxiety and/or depressive disorders, and we were seeking to psychometrically assess the ASAD among patients who matched these criteria, we included patients with at least mild symptoms in our analyses. Therefore, participants who scored less than 10 on the BAI, and less than 5 on the PHQ or less than 14 on the IDS-SR, were ultimately excluded from the EFA and CFA.

### Materials

#### Demographic Variables

Demographic data was collected from all participants, and included age, gender, marital status, educational level and occupational status.

#### Clinical Variables

In order to measure the severity of anxiety symptoms, the BAI was used in both samples. The BAI is a questionnaire comprising 21 items. Each item is rated on a 4-point Likert scale from 0 (not at all) to 3 (severely). The BAI has good psychometric properties: Cronbach’s alpha = 0.94; test–retest reliability: *r* = 0.67; and good convergent and discriminant validity (Fydrich et al., 1992). In order to measure the severity of depressive symptoms, the IDS-SR was used in the GET READY sample. The IDS-SR is a self-report questionnaire, which consists of 30 items. Each item is rated on a 4-point Likert scale from 0 to 3. The IDS-SR has good psychometric properties: Cronbach’s alpha = 0.94; high item total correlations (≥0.50) in 70% of the items; convergent validity = 0.85 (Trivedi et al., 2004; Gili et al., 2010). In the SemCAD sample, the PHQ-9 was used to assess depression severity. The PHQ-9 is a questionnaire comprising nine items. Each item is rated on a 4-point Likert scale from 0 (not at all) to 3 (almost every day). The PHQ-9 is a reliable and valid questionnaire: Cronbach’s alpha = 0.86–0.89; excellent test–retest reliability; high divergent validity; sensitivity and specificity of both 88% for major depression (Kroenke et al., 2001).

#### Development of the Assessment of Self-Management in Anxiety and Depression Questionnaire

In a prior study (van Grieken et al., 2014), a self-management questionnaire was developed for patients with chronic depression. Using the same methods (concept mapping), the authors of the ASAD developed a questionnaire that focused on patients with chronic depression and chronic anxiety disorders.

In phase 1 of concept mapping, patients were asked about helpful self-management strategies they had employed. Two focus groups were formed, consisting of ten and eight patients with chronic anxiety disorders, respectively. In these focus groups, patients completed the statement “I can live with a non-recovered anxiety disorder if….” This technique allowed for self-management strategies to be explored and discussed together. The patients’ responses were then written on a whiteboard and adjusted until they were clear to all participants. This resulted in a total of 176 self-management strategies. Next, when analyzing the findings of these focus groups, any overlapping strategies were subsequently merged. Ultimately, 91 strategies remained. In phase 2 of concept mapping, these 91 strategies were then sent to the participants, who were requested to prioritize and arrange the strategies accordingly. These strategies were then clustered, based on common features, and prioritized by their relevance. In phase 3, the strategies were placed in a concept map and divided into different clusters through the use of the program “Ariadne” (Severens, 2015). In the final phase, the list with the 91 strategies was compared to the existing list of 50 self-management strategies utilized by patients with depression (van Grieken et al., 2014). Once again, any overlapping strategies were removed, and some were merged. This resulted in the Dutch ASAD, which consists of 45 self-management strategies for patients with an anxiety and/or a depressive disorder. Each item can be rated on a 5-point Likert scale, ranging from 1 (not at all) to 5 (very much).

The items in the questionnaire are described in Table 1, including the mean scores of the items, the standard deviations and skewness. The higher the mean score, the higher the application of the selfmanagement behavior. With respect to skewness: 37 of the answer distributions of the 45 items (82%) were symmetrical (scores between −0.5 and 0.5), so we may conclude that the majority of the distributions was not very skewed.

**TABLE 1:** Items in the “Assessment of Self-management in Anxiety and Depression” questionnaire.

What do I do to deal with my anxiety and/or depressive disorder as best as I can:		Mean	SD	Skewness
(1)	Be aware of my thoughts and cognitive biases.	3.08	1.11	0.25
(2)	Acknowledge the signals that tell me I am in danger of relapsing, for example, by using a crisis plan.	2.54	1.12	0.23
(3)	Seek out appropriate treatment.	2.70	1.22	0.29
(4)	Search for information about my anxiety and/or depression.	2.23	1.17	0.80
(5)	Ensure that a professional monitors my medication.	2.38	1.45	0.48
(6)	Continue to seek long-term professional help.	2.58	1.34	0.38
(7)	Make sure I take my medications regularly, by, for example, either keeping them with me at all times or taking them at a specific place or time.	3.26	1.64	−0.36
(8)	Stay physically active, by, for example, taking a walk outside, playing sports or doing housework.	3.10	1.18	0.04
(9)	Keep busy during the day by doing (volunteer) work.	3.01	1.45	−0.08
(10)	Find a (creative) hobby.	2.63	1.25	0.43
(11)	Go outside regularly.	3.18	1.07	0.01
(12)	Use a schedule to stay prepared for upcoming activities.	2.64	1.15	0.24
(13)	Tidy the house.	2.77	1.09	0.38
(14)	Express my feelings by writing in a diary or *via* an online blog.	1.65	1.07	1.63
(15)	Understand what is going on with me.	3.12	1.00	0.14
(16)	Accept my anxiety and/or depression.	2.63	1.09	0.40
(17)	Talk with fellow patients.	1.86	1.00	1.07
(18)	Be in a trusting environment, where I am accepted for who I am.	3.11	1.09	−0.11
(19)	Seek support, by, for example, talking about my anxiety and/or depression with people I trust.	2.64	1.07	0.25
(20)	Explain to people who are important to me that I am suffering from anxiety and/or depression.	2.53	1.12	0.23
(21)	Involve people who are important to me in my treatment.	2.07	1.06	0.83
(22)	Ask others for help.	2.07	0.97	1.05
(23)	Take care of my personal hygiene, by, for example, showering daily and getting dressed.	3.47	1.18	−0.32
(24)	Make sure that I have a good day and night rhythm.	3.16	1.11	−0.01
(25)	Do something for someone else, such as taking care of someone.	2.70	1.14	0.22
(26)	Set goals.	2.46	1.08	0.31
(27)	Honing my attention through meditation, yoga, mindfulness or breathing exercises, for example.	2.03	1.04	0.76
(28)	Be aware of any physical tension in my body, in order to make myself feel relaxed.	2.44	1.06	0.48
(29)	Make sure that I do not take on too many obligations.	2.61	1.15	0.37
(30)	Develop or use a talent.	2.13	1.12	0.72
(31)	Make sure that I establish a good balance between activities and rest.	2.69	1.01	0.31
(32)	Eat and drink healthily.	2.99	1.08	0.25
(33)	Reward myself from time to time, for example, buying something nice.	2.30	1.00	0.73
(34)	Tell myself that better times lie ahead.	2.88	1.15	0.11
(35)	Encourage myself and have perseverance.	3.06	1.11	−0.15
(36)	Take more and more decisions myself, reducing my dependency on others.	2.89	1.17	0.26
(37)	Do my best not to avoid unpleasant situations by facing up to them.	2.64	1.04	0.39
(38)	Encourage myself and put things in perspective.	2.84	1.02	0.27
(39)	Seek out a professional who I have a connection with.	2.61	1.30	0.29
(40)	Distract myself with activities that make me feel good.	3.02	1.09	0.16
(41)	Make sure I avoid things I do not want to do.	2.68	1.06	0.48
(42)	Make sure I have a professional or another important person to take refuge in.	2.67	1.30	0.30
(43)	Keep focused on the present, and stop myself from looking too far ahead.	2.52	1.03	0.40
(44)	Make sure I have the freedom to choose what I do and do not want to do.	2.91	1.04	0.16
(45)	Make sure that I have control by clearly indicating my boundaries.	2.82	0.99	0.36

*This Table comprising the items in the Dutch ASAD has been carefully translated by a professional translator.*

### Data-Analysis

In order to assess the factor structure of the ASAD, both an EFA and CFA were conducted. With EFA, the number of factors can be determined: all measured variables–items–are related to every latent variable (factor). With CFA specification is possible of how each item loads with a unique pattern of zero and non-zero loadings onto hypothesized factors (Matsunaga, 2010). Before running the analysis, the number of factors as well as which measured variable was related to which latent variable was specified in a CFA model.

An EFA with principal axis factoring was conducted to examine the underlying factor structure of the ASAD. To test the internal consistency of the ASAD, Cronbach’s alpha was calculated for each factor, while the communalities (common variance) of the items, factor loadings, and overdetermination (factor to variable ratio) were also examined. In order to assess whether the number of items could be reduced, the correlation matrix of the EFA was inspected for extreme multicollinearity (correlations above 0.80) (Field, 2013). Items with a communality below 0.30, items that cross-loaded on multiple factors with less than 0.2 difference, and items that did not load on any factors were all removed, before the analysis was then subsequently re-run. Given that we anticipated that the factors might correlate, an oblique rotation was chosen (direct oblimin) and the pattern matrix was examined for the factor and item loadings. In order to determine the number of factors that needed to be retained, the scree plot and eigenvalues were inspected. Initially, items with eigenvalues above one were included. The factors deriving from the final factor solution were interpreted and named. The EFA was conducted *via* SPSS, version 26.

Upon completion of EFA, CFA was then conducted to both find evidence for the proposed factor structure in EFA, and to examine whether the factors found *via* EFA were clearly identifiable constructs as measured by the items they contain. To establish the latent structure of items and factors in this way, the CFA was applied with package LAVAAN in R. The input for the CFA were the factors with their items as identified with EFA. The following criteria were used for the goodness-of-fit of the final model, which was based on a reduced number of items: Comparative Fit Index (CFI) and Tucker-Lewis index (TLI) values >0.90, Root Mean Square Error of Approximation (RMSEA) values <0.06 and Standardized Root Mean Square Residual (SRMR) <0.08 (Hooper et al., 2008). Hooper et al. (2008) describe that these fit measures are suitable to determine how well a specified model fits the sample data. Missing data, which pertained to ten missing values distributed over six items, were imputed with case means.

Furthermore, psychometric properties were also examined, including internal consistency, test–retest reliability and discriminant validity. Internal consistency indicates the degree of interrelatedness among the items (Mokkink et al., 2010). This was measured using Cronbach’s alpha, and the sub-scales were assumed to be adequate when Cronbach’s alpha was >0.70 (Terwee et al., 2007). In addition, test–retest reliability was examined using intraclass correlation coefficients (ICC). ICC values were assumed to be excellent if ICC >0.75 (Fleiss, 1986). With respect to the test–retest reliability, there were two observations, as ICC values of at least 0.5 were considered to be acceptable, at least 22 participants were required to complete the retest (Bujang and Baharum, 2017). Discriminant validity concerns the extent to which the factors are diverse and uncorrelated. Therefore, correlations should not exceed 0.7.

## Results

### Participants

The 213 participants in this study comprised 113 participants from the GET READY study and 100 participants from the SemCAD study. In total, 42 participants were excluded from the study because their scores indicated that they had no current symptoms (BAI < 10 and PHQ < 5/IDS-SR < 14). This resulted in 171 participants: 88 from the GET READY study and 83 from the SemCAD study. Table 2 presents the demographic and clinical characteristics of these two samples. The total sample consisted of 103 females and 66 males (the gender of two participants was unknown). Out of the 51 participants from the GET READY sample who were invited to re-take the ASAD after a 2-week period, 43 (84.3%) responded positively, of which the scores of 12 patients indicated that they were in complete remission. Therefore, the test–retest reliability could be examined for 31 participants.

**TABLE 2:** Demographic and clinical variables for each study.

Variables	GET READY sample (*N* = 88)	SemCAD sample (*N* = 83)	*P*
	Mean (SD)	Mean (SD)	
**Demographic variables**			
Age in years	43.4 (12.5)	47.7 (9.0)	**0.01**
Sex, n (%)			0.61
Female	52 (59.1)	51 (61.4)	
Male	36 (40.9)	30 (36.1)	
Missing		2	
Marital status, n (%)			0.86
Single	37 (42.0)	36 (43.4)	
In a relationship	51 (58.0)	47 (56.6)	
Highest educational level, n (%)			**0.02**
Elementary	2 (2.3)	5 (6.0)	
High-school	21 (23.9)	23 (27.7)	
Secondary vocational education	18 (20.5)	30 (36.1)	
Higher professional education or university	45 (51.1)	22 (26.5)	
Other	2 (2.3)	3 (3.6)	
Occupational status, n (%)			**<0.001**
Employed	57 (64.8)	12 (14.5)	
On sick leave	18 (20.5)	42 (50.6)	
Retired	0 (0.0)	4 (4.8)	
Other	13 (14.8)	25 (30.1)	
**Clinical variables**			
Anxiety symptoms (BAI)	11.9 (6.4)	21.8 (10.1)	**<0.001**
Depressive symptoms (IDS-SR)	24.1 (7.6)	–	
Depressive symptoms (PHQ-9)	–	11.5 (5.0)	

*Bolded values indicate statistical significance: p-values lower or equal to 0.01.*

As one can discern in Table 2, there were significant differences between the two samples concerning age, educational level, occupational status and the severity of anxiety symptoms. The SemCAD sample was significantly older, had, on average, a lower educational level, were significantly more likely to be unemployed, as well as experiencing more severe anxiety symptoms. The potential difference between the severity of depressive symptoms could not be statistically tested, as both samples utilized different questionnaires. However, the mean IDS-SR score indicates mild depressive symptoms, while the mean PHQ-9 score indicates moderate depressive symptoms.

### Exploratory Factor Analysis

A principal axis factor analysis was conducted on the 45 items of the ASAD with oblique rotation (direct oblimin). The Kaiser-Meyer-Olkin (KMO) measure verified the sampling adequacy of the analysis, KMO = 0.85 [“meritorious” according to Hutcheson and Sofroniou (1999)] and all KMO values for individual items were greater than 0.54, which is above the acceptable limit of 0.5 (Field, 2013). Bartlett’s test of sphericity (Bartlett, 1951) was statistically significant (*p* < 0.001). Both tests supported the factorability of the correlation matrix. An initial analysis was run to obtain eigenvalues for each factor in the data. This resulted in 13 factors exceeding Kaiser’s criterion of eigenvalue one, the combination of which explained 67.87% of the variance. Of those 13 factors, three had an eigenvalue above two. The scree plot was ambiguous and showed inflections that would justify retaining either three or five factors. We opted to retain three factors because this provided the most interpretable solution. Parallel Analysis resulted in seven factors that were not interpretable in a clinically meaningful way; three of the factors in the parallel analysis corresponded to the factors had adjusted eigenvalues >1) and could be interpreted. So, also based in these findings it was decided to retain three factors.

Items with factor loadings larger than 0.3 were included in the factor. In total, 24 items were excluded from the analysis, due to the fact that they did not load on any factor (*N* = 7), showed cross-loadings between factors with less than 0.2 difference (*N* = 6), or had a communality below 0.3 (*N* = 11). Thus, the number of items was reduced from 45 to 21 after the analysis. The 21-item version of the ASAD, the ASAD-Short Form (SF), explained after rotation 51.27% of the variance, with factor one contributing 30.08%, factor two contributing 12.45%, and factor three contributing 8.74%.

Close inspection of the items that were grouped into the same factor (see Table 3) led to the factors being named as follows: factor 1 represents Seeking support; factor 2 represents Daily life strategies; and factor 3 represents Taking ownership. More information about the factors can be found in Table 3.

**TABLE 3:** Explanation of the underlying factors.

Factors	Name	Explanation
Factor 1	Seeking support	Seeking and maintaining treatment or support from a professional and significant others
Factor 2	Daily life strategies	Active lifestyle, taking care of yourself, finding balance, structure, setting goals and making plans to achieve them
Factor 3	Taking ownership	Taking ownership and control, having focus, intention and awareness

### Confirmatory Factor Analysis

After EFA, CFA was conducted to examine whether the factors found with EFA were clearly identifiable constructs as measured by the items they contain. This should be expressed in at least moderate goodness-of-fit measures for the estimated model with three (correlated) factors. With the 21 items corresponding to the three factors found *via* EFA as input, CFA resulted in moderate fit measures: χ^2^ = 439.35 (df = 168), CFI = 0.80, TLI = 0.78, RMSEA = 0.09 (CI 0.08–1.00), SRMR = 0.09.

Correlation of the factors was permitted, the following values were found: R seeking support-daily life strategy = 0.49, R seeking support-taking ownership = 0.47, R daily life strategy -taking ownership = 0.39.

### Internal Consistency

To assess the internal consistency of the factors, the communalities (common variance) of the items, overdetermination (factor to variable ratio), and factor loadings were examined. Starting with the communalities, the 21-item ASAD-SF had four items with communality above 0.50 and a mean communality of 0.44, which is relatively low. Overdetermination of factors was high, as high loadings were shown on at least three items for each factor (MacCallum et al., 1999). Therefore, despite low communalities, good recovery of factors was still achieved (MacCallum et al., 1999). Continuing with the factor loadings, factor 1 had five factor loadings above 0.60 and two factor loadings above 0.50, factor 2 had four factor loadings above 0.60 and two factor loadings above 0.50, while factor 3 had three factor loadings above 0.60. Therefore, factor 1 and 2 had five or more strongly loading items (0.50 or better), thus indicating solid factors (Costello and Osborne, 2005). Although factor 3 had only three items, these items could nevertheless still be interpreted in a meaningful way, and, indeed, three items can still indicate a solid factor (Costello and Osborne, 2005). Cronbach’s alpha was calculated for the 21-item ASAD-SF, resulting in high internal consistency, α = 0.88. All sub-scales also had high internal consistency (factor 1: α = 0.88, factor 2: α = 0.86, factor 3: α = 0.76). Factor loadings found with EFA and CFA respectively are presented in Table 4.

**TABLE 4:** Factor loadings per item of the ASAD questionnaire.

		Exploratory factor analysis			Confirmatory factor analysis		
		Factor loadings			Factor loadings		
		Seeking support	Daily life strategies	Taking ownership	Seeking support	Daily life strategies	Taking ownership
(6)	Continue to seek long-term professional help.	0.796			0.582		
(3)	Seek out appropriate treatment.	0.650			0.648		
(5)	Ensure that a professional monitors my medication.	0.635			0.506		
(22)	Ask others for help.	0.622			0.720		
(39)	Seek out a professional who I have a connection with.	0.612			0.618		
(4)	Search for information about my anxiety and/or depression.	0.590			0.570		
(42)	Make sure I have a professional or another important person to take refuge in.	0.519			0.696		
(19)	Seek support, by, for example, talking about my anxiety and/or depression with people I trust.	0.472			0.600		
(20)	Explain to people who are important to me that I am suffering from anxiety and/or depression.	0.439			0.633		
(21)	Involve people who are important to me in my treatment.	0.392			0.574		
(8)	Stay physically active, by, for example, taking a walk outside, playing sports or doing housework.		0.722			0.653	
(24)	Make sure that I have a good day and night rhythm.		0.712			0.624	
(11)	Go outside regularly.		0.711			0.714	
(26)	Set goals.		0.622			0.688	
(28)	Be aware of any physical tension in my body, in order to make myself feel relaxed.		0.576			0.607	
(32)	Eat and drink healthily.		0.574			0.552	
(10)	Find a (creative) hobby		0.447			0.574	
(12)	Use a schedule to stay prepared for upcoming activities.		0.402			0.573	
(45)	Make sure that I have control by clearly indicating my boundaries.			0.768			0.850
(44)	Make sure I have the freedom to choose what I do and do not want to do.			0.715			0.717
(43)	Keep focused on the present, and stop myself from looking too far ahead.			0.600			0.574

### Test–Retest Reliability

Test–retest reliability over a 2-week period was excellent (factor 1: ICC = 0.76, 95% CI 0.49–0.89; factor 2: ICC = 0.88, 95% CI 0.71–0.95; factor 3: ICC = 0.86, 95% CI 0.72–0.93).

### Discriminant Validity

As one can see in Table 5, the Seeking support factor correlated with both the Daily life strategies (0.31) and the Taking ownership factor (0.31). Furthermore, the Daily life strategies factor also correlated with the Taking ownership factor (0.28). The fact that these correlations are well below 0.7 indicates that the variables in the factors were more strongly related to their own factors than other factors.

**TABLE 5:** Factor correlation matrix.

	Seeking support	Daily life strategies	Taking ownership
Seeking support	1.000		
Daily life strategies	0.310	1.000	
Taking ownership	0.309	0.280	1.000

## Discussion

### Main Findings

This study represents the first attempt to evaluate the ASAD questionnaire by performing EFA and CFA, in order to establish the underlying factor structure, psychometric properties, and the possibility of reducing items without sacrificing on precision. Three consistent factors were identified: Seeking support, Daily life strategies, and Taking ownership. Seeking support contained ten items that pertained to seeking and maintaining support from both professionals and significant others. Daily life strategies contained eight items related to maintaining an active lifestyle, healthy habits and engaging in structured activities. Taking ownership contained three items, which pertained to taking control over one’s life and focusing one’s attention on recovery. The number of items in the questionnaire was subsequently reduced from 45 to 21. The results thus support the psychometric properties of a short form (SF) of the ASAD. The internal consistency of the 21-item ASAD-SF as well as their sub-scales was high, test–retest reliability indicated excellent reliability on all factors, and discriminant validity was also shown.

### Comparisons to Previous Literature

Of those studies that have psychometrically evaluated similar self-management questionnaires, Coulombe et al. (2015) and Smith et al. (2017) research are particularly worthy of comparison to this study.

The factors identified in this study are similar to the subscales found in Coulombe et al. (2015) study. They examined the Mental Health Self-Management Questionnaire (MHSQ), which is a 64-item questionnaire developed for patients who are recovering from depression, anxiety or bipolar disorders. The subscale Clinical appears to be similar to the factor Seeking support. However, the clinical subscale does not include support from significant others, whereas the Seeking support factor includes both professional support and support from significant others. The subscale Vitality resembles the factor Daily life strategies, insofar as both focus on engaging in healthy activities. Moreover, the subscale Empowerment bears some similarities with the factor Taking ownership. However, the Empowerment subscale is more extensive, and contains items like “loving myself as I am” and “congratulating myself for my successes.” However, the MHSQ contains almost three times as many items as the 21-item ASAD-SF, and, hence, is far more burdensome for patients to complete.

There was also some overlap between our study and the Partners in Health (PIH) scale. The PIH scale is an Australian questionnaire, which focuses on the self-management strategies employed by the chronically ill. Smith et al. (2017) conducted a CFA to establish the factors of the PIH. When comparing the ASAD-SF to the PIH scale, the same similarities and differences appear as with the MHSQ: the factor Patient-health professional partnership is similar to the factor Seeking support, albeit it only focuses on professional support, as opposed to support from significant others too. The factor Coping with a chronic illness is similar to the factor Daily life strategies, insofar as both focus on staying active. The similarities between, on the one hand, the identified factors of the ASAD-SF, and the PIH-factors Knowledge of illness and treatment and Recognition and management of symptoms, on the other, were less obvious, potentially because the PIH scale focuses primarily on self-management of physical illness rather than mental illness. Although, of course, there are many similarities between physical and mental illness, not to mention that the underlying constructs of handling a chronic disease might be comparable, one would expect to see differences between the PIH and the ASAD-SF.

van Grieken et al. (2014, 2015), Villaggi et al. (2015), and Schreurs et al. (1988) all conducted studies that qualitatively established clusters, dimensions and factors related to self-management.

Given that the ASAD was developed as an expansion of the self-management strategies identified by van Grieken et al. (2014), it is altogether understandable that the factors found in our factor analyses match the sub clusters found by these authors. The sub clusters identified by van Grieken et al. (2014, 2015) were clustered using concept mapping. While the authors psychometrically evaluated the 50 self-management strategies that were identified by their participants, no satisfactory EFA could be performed (van Grieken et al., 2018). Our study did partially confirm the sub clusters identified in their studies. More specifically, the factor Seeking support is in line with the sub clusters Active coping with professional treatment and Social engagement (van Grieken et al., 2014). Furthermore, the factor Daily life strategies appears to be in accordance with the sub clusters Active self-care, structure and planning, Daily life strategies and rules, Engaging in activities, and Structured attention to oneself (van Grieken et al., 2014, 2015). In contrast, no sub clusters were described that matched the factor Taking ownership.

There are also similarities between the present study and the factors identified in Villaggi et al. (2015) study. In their study, self-management strategies were explored among a sample of patients with anxiety disorders, depressive disorders and bipolar disorders. The factor Seeking support resembles the dimension Surrounding myself with people who make me feel better, although this dimension is only focused on informal support, rather than also on professional support. The factor Daily life strategies is in accordance with both the dimension Functional (e.g., Creating a routine and taking action) and the dimension Physical (e.g., Maintaining a healthy lifestyle and Managing one’s energy levels). The factor Taking ownership is in accordance with the sub dimension Empowering oneself. This study from Villaggi et al. further revealed that patients with anxiety, depressive and bipolar disorders utilized similar self-management strategies. This indicates that an approach that is focused on affective disorders might be appropriate for self-management (Coulombe et al., 2016).

Finally, the factors identified in this factor analysis can be compared with the Utrecht Coping List (Schreurs et al., 1988). The Utrecht Coping List is a 47-item questionnaire that encapsulates seven domains of coping. The domain Seeking social support appears to be very similar to our factor Seeking support. Moreover, the domain Active problem solving is in line with our factor Taking ownership. The other domains were not concordant with the factors found in this factor analysis, most likely because coping is not the same as self-management; while self-management focuses on how one handles a specific disease, coping is more focused on coping with problems in general.

When comparing our findings to the six core self-management skills defined by Lorig and Holman (2003), it appears that the skill Formation of a patient-provider partnership overlaps with our factor Seeking support. In addition, the skill Action planning bears some resemblance to our factor Daily life strategies. The other four self-management skills (Problem solving, Decision making, Resource utilization and Self-tailoring) appear to be intertwined in our three factors.

To conclude, most of the overlap between our findings and extant literature pertained to the factors Seeking support and Daily life strategies. Within other self-management questionnaires, the factor Seeking support is primarily focused on seeking support from a professional (Coulombe et al., 2015; Smith et al., 2017), and, indeed, no factors that combined both professional and informal support were found in extant literature. Factors similar to Daily life strategies were often identified (van Grieken et al., 2014, 2015; Coulombe et al., 2015; Villaggi et al., 2015; Smith et al., 2017). Although less pronounced, we also found a degree of overlap between our third factor Taking ownership, albeit these similar factors focused on empowerment (Coulombe et al., 2015; Villaggi et al., 2015). We did not identify any other important factors within extant literature that were not found in our study.

### Strengths and Limitations

There are several strengths to the present study. First, this study is the first to statistically examine the ASAD, and thus provide insight into the underlying factor structure, internal consistency, test–retest reliability and discriminant validity of this questionnaire. Moreover, this study sheds light on how those items that are grouped together are subsequently translated into self-management styles. The 21-item ASAD-SF has proven to be an internally consistent and reliable measure for assessing self-management strategies among a sample of Dutch patients with (chronic and partially remitted) anxiety and depressive disorders.

The second strength is that the questionnaire focuses on patients suffering from both anxiety and depressive disorders. This is important, because most studies focus solely on either patients with anxiety disorders or patients with depressive disorders, despite the fact that previous research has shown that anxiety and depressive disorders are highly comorbid (Lamers et al., 2011). Comorbid anxiety and depressive disorders increase chronicity (Penninx et al., 2011), which is one of the reasons for encouraging modes of self-management.

The findings of this study should be interpreted with several limitations in mind. First, the sample of 171 patients is a relatively small sample for factor analysis (Tabachnick and Fidell, 2012). However, sample size is also dependent on factor loadings and communalities (Field, 2013). Matsunaga (2010) argues that when items are closely related to a factor, a sample size between 100 and 200 is sufficient. Our sample of 171 cases should suffice, especially since this study concerns a first exploration of factors. As the results demonstrated, the internal consistency of the ASAD-SF and its three factors is high. Although the communalities show less evidence of internal consistency, the factor loadings and overdetermination indicate that the factors are internally consistent. Furthermore, the preliminary analysis did indicate that the sample was appropriate for factor analysis. The advantage of a larger sample size is that it would have allowed us to split the sample, and conduct EFA on one sample, while conducting CFA on the other sample, which, in turn, would result in a more stringent assessment of the factor structure (Wang et al., 2013). However, this study did not aim to fit the data to suit the EFA and CFA, but rather aimed to provide insight into the covariances between factors, and, in turn, into the factors of the newly developed ASAD.

The second limitation of this study pertains to the use of two different datasets. These datasets were merged to increase the sample size, in order to be able to conduct the EFA and CFA. The participants in this study were screened for depression *via* two different questionnaires, the PHQ-9 and the IDS-SR, which might be less reliable than using a single screening questionnaire. However, both the PHQ-9 and the IDS-SR have been found to be reliable and valid questionnaires for assessing depression. Moreover, these questionnaires were only used to assess the severity of symptoms, in order to exclude those who had no symptoms. Nevertheless, using two datasets could well result in a greater level of heterogeneity. In the SemCAD sample, patients experienced, on average, moderate anxiety and depressive symptoms, while in the GET READY sample the anxiety and depressive symptoms were mild. One explanation for this is that the SemCAD sample comprised patients with chronic anxiety and/or depressive disorders, while the GET READY sample consisted of patients who were in partial remission from anxiety and/or depressive disorders.

### Implications for Practice and Research

Several implications for practice can be suggested. First, this study provides a feasible self-management questionnaire that can be offered to patients with (chronic or partially remitted) anxiety and depressive disorders. The use of self-management strategies by patients is likely to be cost-effective for both the prevention and treatment of anxiety and depressive disorders, and could reduce the burden of disease, as well as the economic burden of mental health disorders. The results of this study could also provide guidance for patients regarding which specific self-management strategies to focus on. For example, they could self-manage their disorder by seeking support from professionals and important others (Seeking support), by maintaining a healthy lifestyle and engaging in activities (Daily life strategies), or by taking control and staying focused on their recovery (Taking ownership). Second, the three factors identified in this study could also be considered in the development of relapse prevention programs. With respect to E-health modules, attention should be paid to the themes Seeking support, Daily life strategies and Taking ownership. For example, information on how to engage with important others and MHPs could help patients to seek out support. Third, professionals could advise patients in how best to execute these self-management themes and strategies. One possible approach might be for patients to complete the ASAD-SF during their actual treatment. If they scored low on one or more factors, then this could help guide professionals in advising which self-management strategies to utilize. For example, if patients scored low on the factor Seeking support, the specific attention could be paid to how patients can better involve professionals and important others in their recovery. Fourth, depending on the purpose of completing the ASAD, patients and professionals could choose which version to use. If a detailed description of self-management strategies is required, then patients could complete the 45-item ASAD. Alternatively, if a shorter version is preferred that provides insight into important clusters of self-management, then patients could complete the 21-item ASAD-SF.

Given that this study indicated that the 21-item ASAD-SF is appropriate, this version should be further explored and validated among a sample of patients with (chronic or partially remitted) anxiety and depressive disorders. Alongside this, to increase generalizability, more studies are required to examine the English version of the ASAD within other settings and countries.

## Conclusion

This study presents an initial test of a questionnaire that assesses self-management strategies for patients with chronic or partially remitted anxiety and/or depressive disorders. In this respect, the ASAD-SF demonstrated good psychometric properties. The statistical evaluation conducted here constitutes the first examination of factors of self-management strategies among this sample, and resulted in a stable 3-factor structure. The 21-item ASAD-SF could be offered to patients with (chronic or partially remitted) anxiety and depressive disorders, with the identified factors providing guidance to patients and professionals concerning which specific strategies to focus on. Ultimately, these factors could be incorporated into the development of new relapse prevention programs.

## Data Availability Statement

The data analyzed in this study is subject to the following licenses/restrictions: Data from the SemCAD study cannot be made publicly available due to institutional restrictions. Data from the GET READY study can be made available upon request. Requests to access these datasets should be directed to EK-D, esther.krijnendebruin@inholland.nl.

## Ethics Statement

The studies involving human participants were reviewed and approved by The Medical Ethics Committee of the Amsterdam UMC, location VU University Medical Center and the Institutional Review Board of the University Medical Center Utrecht. The patients/participants provided their written informed consent to participate in this study.

## Author Contributions

EK-D, AM, AE, AS, HS, JS, NB, and BM contributed to conception and design of the study. EK-D and SD performed the statistical analysis. EK-D wrote the first draft of the manuscript. AE and SD wrote sections of the manuscript. All authors contributed to manuscript revision, read, and approved the submitted version.

## Conflict of Interest

The authors declare that the research was conducted in the absence of any commercial or financial relationships that could be construed as a potential conflict of interest.

## Publisher’s Note

All claims expressed in this article are solely those of the authors and do not necessarily represent those of their affiliated organizations, or those of the publisher, the editors and the reviewers. Any product that may be evaluated in this article, or claim that may be made by its manufacturer, is not guaranteed or endorsed by the publisher.

## Abbreviations

ASAD: Assessment of Self-management in Anxiety and Depression
ASAD-SF: Assessment of Self-management in Anxiety and Depression-Short Form
EFA: exploratory factor analysis
CFA: confirmatory factor analysis
CFI: Comparative Fit Index
TLI: Tucker Lewis Index
RMSEA: Root Mean Square Error of Approximation
SRMR: Standardized Root Mean Square Residual
MHSQ: Mental Health Self-Management Questionnaire
PIH: Partners in Health
BAI: Beck Anxiety Inventory
IDS-SR: Inventory of Depressive Symptomatology–Self Report
PHQ-9: Patient Health Questionnaire-9
ICC: intraclass correlation coefficients
KMO: Kaiser-Meyer-Olkin.
